# Analysis of Reference Ranges of Total Serum Protein in Namibia: Clinical Implications

**DOI:** 10.3390/proteomes8020007

**Published:** 2020-04-15

**Authors:** Josephine N. Henok, Benjamin I. Okeleye, Elizabeth I. Omodanisi, Seteno K. O. Ntwampe, Yapo G. Aboua

**Affiliations:** 1 Department of Health Sciences, Faculty of Health and Applied Sciences, Namibia University of Science and Technology (NUST), Private Bag 13388, Windhoek, Namibia; josephinendeuza15@gmail.com (J.N.H.); yaboua@nust.na (Y.G.A.); 2 Bioresource Engineering Research Group (BioERG), Department of Biotechnology, Faculty of Applied Sciences, Cape Peninsula University of Technology, P.O. Box 652, Cape Town 8000, South Africa; lizzy.omodanisi@gmail.com (E.I.O.); karabo.ntwampe@nwu.ac.za (S.K.O.N.); 3 School of Chemical and Minerals Engineering, North-West University, Private Bag X1290, Potchefstroom 2520, South Africa

**Keywords:** clinical laboratory, non-parametric, reference range, total serum protein

## Abstract

A reference range is an essential part of clinical laboratory test interpretation and patient care. The levels of total serum protein (TSP) are measured in sera to assess nutritional, liver, and kidney disorders. This study determined the TSP reference range with respect to gender, age, and region in Namibia. A retrospective cross-sectional study was conducted to determine the TSP reference range among 78,477 healthy participants within the ages of less than one year to more than 65 years in 14 regions of Namibia. The reference range of TSP was 51–91 g/L for females and 51–92 g/L for males. A reduced TSP range of 48.00–85.55 g/L (2.5–97.5 percentiles) was established at <1–5 years and increased towards adolescence. An uttermost range of 54–93 g/L was observed from 36–65 years of age. At the age >65 years; a steady decline in the reference range (51.00–89 g/L) was recorded. An upper TSP range of 53–92 g/L (2.5–97.5 percentiles) was detected in Erongo, Zambezi, Hardap, Kavango East, and a comparable trend was also seen in Omusati with a 54–91 g/L range. Meanwhile; a reduced TSP range of 50–89 g/L was identified in Ohangwena. This study showed that gender, age, and geographical location can impact TSP levels with a significant clinical difference (*p* < 0.05) between each category.

## 1. Introduction

Total serum proteins (TSP) are measured in the body to diagnose nutritional challenges such as protein energy wasting (PEW), which is the state of decreased body stores of protein and energy. This can be caused by the reduced intake of protein and energy-rich food and occurs during malnutrition [1]. Albumin constitutes 65% of the TSP present in the blood and it is responsible for the transport of substances such as unconjugated bilirubin and some hormones. It is responsible for the maintenance of the 80% colloid osmotic pressure in the blood and is used as a long-term indicator for malnutrition; hence, the diagnoses of nutrition-related chronic deficiencies [2]. TSP is used as a liver function test to support the diagnosis of liver disorders. The disorders can stimulate the increase or inhibit the production of the liver’s total plasma protein, meanwhile, the TSP levels are usually decreased in conditions that are commonly associated with liver dysfunction. Some disorders that are associated with high TSP levels include human immunodeficiency virus (HIV) and hepatitis [3]. TSP levels are also measured as part of the kidney function tests. Disorders such as glomerulonephritis are associated with decreased levels of TSP as a result of damage to the glomeruli; therefore, affecting the filtration that leads to excretion of protein into the urine. The loss of proteins in the urine then leads to decreased levels of proteins in the blood. An example of a kidney disorder that is associated with high TSP levels is kidney inflammation [4].

Protein levels in the human body have different ranges depending on age, ethnicity, and gender. This was revealed in a study conducted in Hawaii among four different races, which indicated a wider reference range variation among the different population groups [5]. Scientific reports have shown the effects of dietary protein intake, gender, and age on TSP, and that it decreases as the person ages and the rate of decrease is more rapid in women than men, with a suggestion that an appropriate range of TSP based on age and gender differences should be used for clinical diagnostic applications [6,7].

Reference ranges are sets of values used by health professionals to interpret patient results obtained from the laboratory. Reference ranges are considered as the authoritative tools for the interpretation of data and decision making in laboratory science. It is established by collecting samples from a population that is considered to be normal or healthy [8]. The collected samples are tested for the concentration of the different analytes, e.g., sodium, glucose, proteins, enzymes, and many other laboratory analytes. The results obtained are then used to determine a range that lies within the 95% confidence interval on the Gaussian distribution curve. Proteins are important in the body because they are responsible for the growth and repair of tissues. Proteins are also responsible for the transport of substances into the body, can act as enzymes, and play a role in fighting against infections (i.e., antibodies). Some proteins help in the development of structures in some organelles such as microfilaments and microtubules [9]. The direct method used in the determination of reference range of TSP is expensive, difficult to perform, often inaccurate, and non-reproducible. This makes it difficult for most laboratories to have reference ranges of their own; hence, indirect methods have been used to determine the reference ranges [3]. Reference intervals are established by selecting statistically and sufficiently healthy subjects (a minimum of 120 subjects) [3,10]. Indirect methods increase the accuracy of the reference range compared to the direct method, and to minimize the risk of inaccuracy and costs, it is recommended that about 20 samples should be used by clinical laboratory to represent the healthy population to verify the reference ranges that have been established [3,10]. However, different indirect methods are available such as the parametric method and non-parametric method. The non-parametric method is more accurate because it tends to use complex statistical algorithms to derive the final intervals as well as Chauvenet criteria, which statistically detects and rejects outliers or bad data especially when the probability of occurrence is less than 1/(2n), where *n* is the number of measurements in the data pool [11,12]. Reference ranges are determined to differentiate healthy from non-healthy persons and to detect the specific causes of illnesses. They are also used in the monitoring of patients in disease progression or drug therapy after illnesses and prognostic factors of the correct therapy for the patients [3].

To the best of our knowledge, the investigation into the reference ranges of TSP has never been conducted before in Namibia. Thus, this study focused on the determination of TSP reference ranges according to gender, age groups, and regions in Namibia. An automated dry chemistry analyzer was used for the TSP analysis and the TSP reference range was established using the indirect non-parametric percentile technique.

## 2. Materials and Methods

### 2.1. Study Design and Subjects

This study was a retrospective, descriptive cross-sectional examination, which involved the analysis of pre-tested data for the period of one year; June 2017–June 2018. We evaluated 78,477 healthy participants within the age group of < 1 – > 65 years and covering 14 regions in Namibia. It focused on the analysis of the reference range of TSP data using indirect non-parametric method and in comparison to gender, age groups, and regions in Namibia. Permission to carry out the study was sought and granted by the Department of Health Sciences, Namibia University of Science, and Technology (NUST), Ministry of Health and Social Services (MoHSS), and by Namibia Institute of Pathology (NIP) with ethical approval and protocol number JH-2018 being obtained.

### 2.2. Sampling and Data Collection Techniques

Convenient and stratified sampling methods were used, whereby subjects were selected because of availability and the data was divided according to gender, age groups, and region. Data did not contain the patient’s name for confidentiality. The laboratory clinic had a fully automated dry chemistry analyzer (Abbott architect), which was used for the TSP analysis. The maintenance, calibration, and quality control records of the equipment during the period of the study were confirmed. The instrument uses the Biuret method to quantify the amount of TSP present in a sample. The Biuret reagent contains copper ions that bind to peptide bonds present in the sample. The reaction gives-off a chromogenic solution with the intensity being directly proportional to the concentration of the TSP present in the blood. It further uses the molecular absorption spectrophotometry to measure the absorbance of the samples, which was converted to concentration in g/L [13].

### 2.3. Data Analysis

Descriptive statistics were used to analyze the collected data and to establish the non-parametric percentile limits. The 2.5th percentile was the lower limit and 97.5th percentile was the upper limit. The data was analyzed using SPSS version 25 and Graph Pad Prism version 5. The frequency, mean, standard deviation, and percentiles were generated. Box plots were visually inspected and the outliers were removed using the boxplot function. Outliers are assumed to be false beyond the lower and higher quartile. Meanwhile, the data were log-transformed because of the level of skewness. ANOVA was also performed to compare the mean values of results between the gender, age groups, and regions. A *p*-value of < 0.05 was considered to be statistically significant. The reliability and validity of the findings were initially determined using Equation (1):

Fisher skewness coefficient = (Skewness/Standard (Std.) Error of Skewness),(1)

Because of the extreme sensitivity of Fisher measurement of skewness to outliers, Equation (2) was mainly used:

(2) $$\mathrm{Pearson}\;\mathrm{skewness}\;\mathrm{coefficient}\;({\mathrm{Sk}}_{p})\;=\;3[(\bar{x}\;-\;\mathrm{Md})/\mathrm{SD}],$$

where: $\bar{x}$ = mean value, Md = median and SD = standard deviation.

## 3. Results

### 3.1. Analysis of the Subjects

Out of 81,509 recruited in this study, 78,477 participants were further analyzed after 3.72% outliers were detected and removed. There were 44,845 (57.1%) females and 33,632 (42.9%) males subjects with a male to female ratio of 1:1.3. The number and percentage of the subjects stratified according to age group were as follows: <1 year, 3.3% (N = 2549); 1–5 years, 5.4% (N = 4257); 6−12 years, 3.5% (N = 2709); 13−18 years, 4.0% (N = 3152); 19−25 years, 10.2% (N = 8027); 26−35 years, 22.9% (N = 18003); 36−45 years, 16.9% (N = 13243); 46−55 years, 12.6% (N = 9867); 56−65 years, 9.5% (N = 7420); and >65 years, 11.8% (N = 9250). Data collected were representing 14 geopolitical region of Namibia with the percentage of subjects recorded for Otjozondjupa, 3.7% (N = 2925); Khomas, 33.2% (N = 26014); Oshikoto, 7.7% (N = 6021); Oshana, 15.2% (N = 11887); Erongo, 3.8% (N = 2952); Ohangwena, 5.2% (N = 4114); Kavango West, 1.3% (N = 979); Zambezi, 3.2% (N = 2523); Karas, 2.4% (N = 1862); Kunene, 2.1% (N = 1681); Kavango East, 15.7% (N = 12353); Omusati, 3.8% (N = 2986); Omaheke, 0.8% (N = 648); and Hardap, 2.0% (N = 1532).

### 3.2. Reference Range for Total Serum Protein

The overall reference range for TSP from the 2.5^th^–97.5^th^ percentile observed in this study was 51–91 g/L, as shown in Table 1. The results were presented in parametrics such as mean ± standard deviation (SD) and the overall distributions were statistically significant (*p*-value < 0.05) as regards to the mean of their clinical values. Meanwhile, the reference ranges were obtained based on the non-parametric method of 2.5th–97.5th percentile interval, which is convenient for a Gaussian and non-Gaussian distribution. The skewness was observed at −0.229 with a standard error of 0.009 as represented in Table 1. Moreover, the distribution was asymmetric and negatively skewed with a long left tail and Fisher skewness coefficient (Skewness/Std. Error of Skewness) of −25.44, compared with −1.96 to +1.96 considered to be of insignificant difference from a normal distribution, which is supposed to be 0. However, the Pearson skewness coefficient (Sk_p_) of −0.13 was observed (Sk_p_ = 3[($\bar{x}$ − Md)/SD]), which was within −0.5 to +0.5, a suggested and generally acceptable skewness.

This discrepancy was due to the extreme sensitivity of the Fisher measure of skewness to outliers. Meanwhile, these negative coefficients indicated a negative level of skewness, whereby the mean (72.58) is smaller than the median (73.00), which is directly opposite for the Pearson skewness coefficient. Sk_p_ with 0 value indicated an equal mean and median, thus symmetric in its distribution. Sk_p_ of −0.13 fell within the acceptable values (±0.5); therefore, showed that the distribution was asymmetric and not unduly skewed with the high level of reliability or validation of the reference range for TSP recorded; hence, the analysis of kurtosis, which uses the same formula and principle as skewness. Kurtosis was used to measure the extent to which observations cluster around a central point. The kurtosis was observed at −0.159 with a standard error of 0.017 (Table 1). Fisher kurtosis coefficient (Kurtosis/Std. Error of Kurtosis) of −9.35 was noted. This also falls outside the acceptable limit of ±1.96; thus, indicating that the distribution was platykurtic with data values flattered and more dispersed along the *X*-axis (negative kurtosis) than a normal distribution.

### 3.3. Gender-Based Reference Range and Data Distribution

The reference range for female and male were 51–91 g/L and 51–92 g/L, respectively (Table 2), with a significant clinical difference (*p* < 0.05). The gender distribution skewness was observed at −0.215 and −0.253 with a standard error of 0.012 and 0.013 for females and males, respectively (Table 2). Negatively skewed and asymmetric values were obtained as the Fisher skewness coefficient (Skewness/Std. Error of Skewness) of −17.92 (female) and −19.46 (male) were not within the standard range (±1.96). However, the Sk_p_ coefficient of −0.22 and −0.30 was observed for females and males respectively, in this study, which fell within ±0.5 acceptable skewness. Meanwhile, the kurtosis for females was observed at −0.129 (standard error 0.023), while for males it was at −0.191 with a standard error of 0.027 (Table 2). Fisher kurtosis coefficient (Kurtosis/Std. Error of Kurtosis) of −5.61 and −7.07 were observed for females and males, respectively. These fell outside the acceptable limit and normal distribution of ±1.96 and 0, respectively; which signified that the distribution was platykurtic with data values being flattered and more dispersed along the *X*-axis.

### 3.4. Reference Range and Data Distribution According to Age Group

**Figure 1:**
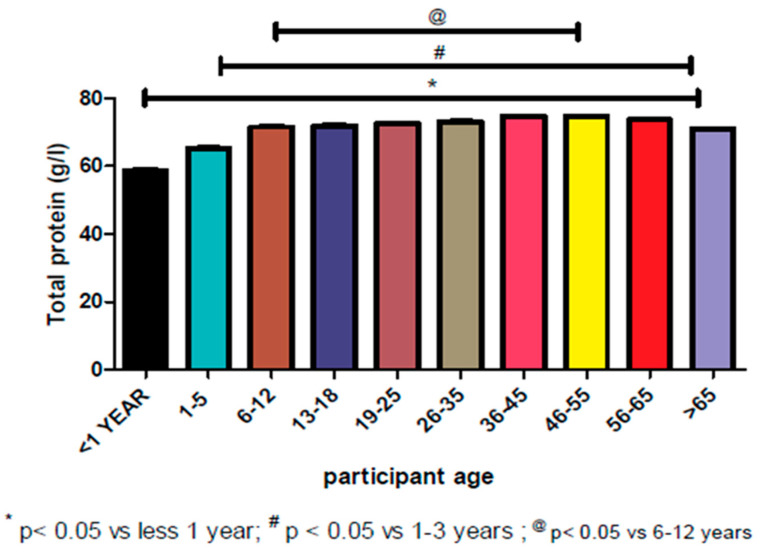
Distribution of total protein according to age groups in relation to statistical significant difference.

A lower TSP range of 48–85.55 g/L (2.5–97.5 percentiles) was observed at <1–5 years of early age and increased towards adolescence as shown in Table 3. A peak range of 54–93 g/L was noted from 36–65 years of age (Table 3) when compared to the overall range of 51–91 g/L (Table 1). At the age >65 years, a steady-state of decline in reference range (51–89 g/L) was recorded. The mean values followed a similar pattern. Negative skewness and kurtosis values were predominant except for age <1 year with 0.688 and 0.141 for skewness and kurtosis, respectively. The same was noticed for kurtosis with 0.219 and 0.176 values for 46–55 and 56–65 years age groups (Table 3). Furthermore, the Sk_p_ coefficient was between −0.37 – 0.20 for age group range of 1–5 to >65 years, which fell between the acceptable skewness of the symmetrical distribution of ±0.5, except for <1 age group value that was highly skewed with Sk_p_ coefficient of 1.13, which fell out of an acceptable value of skewness. However, all values were asymmetrical, except for age groups 1–5, 46–55, and 56–65 years that were closer to the symmetrical distribution of zero (0) with Sk_p_ coefficient values of 0.20, −0.12, and −0.12. Meanwhile there was a significant clinical difference between the values (*p* < 0.05), as shown in Figure 1.

### 3.5. Reference Range Based on Geographical Location and Symmetric Data Analysis

**Figure 2:**
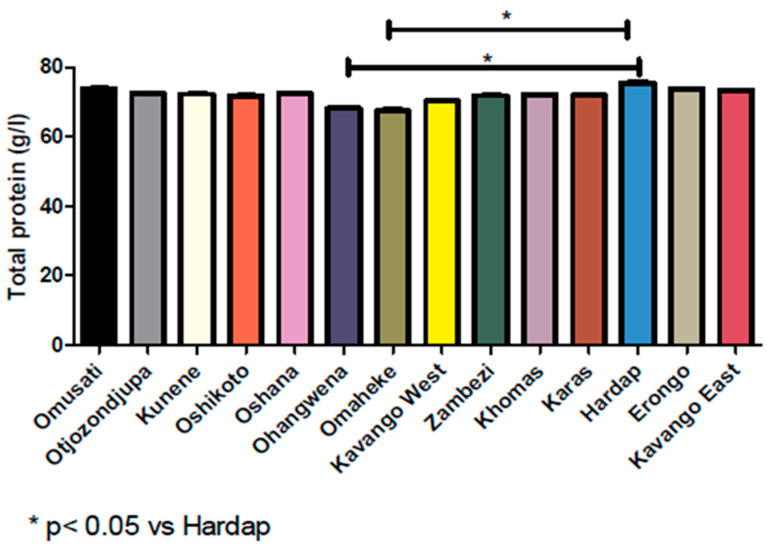
Distribution of total protein according to geographical region in relation to statistical significant difference.

A higher TSP range of 53.00–92.00 g/L (2.5–97.5 percentiles) was observed in Erongo, Zambezi, Hardap, Kavango East, and a similar trend in Omusati with 54.00–91.00 g/L range (Table 4). Meanwhile, a lower TSP of 50.00–89.00 g/L was detected in Ohangwena when compared to the overall range of 51–91 g/L (Table 1). The mean values followed similar patterns. Negative skewness of −0.011–−0.342 was recorded for all the regions which are similar to kurtosis values (−0.099–−0.266) except for Erongo, Kunene, and Omusati with kurtosis value of 0.021, 0.028, and 0.051, respectively (Table 4). Moreover, the Sk_p_ coefficient was noted between −0.35–0.11, which falls between the acceptable skewness of the symmetrical distribution of ±0.5, except for the Omaheke value that is highly skewed with Skp coefficient of 0.60, which falls out of the acceptable value of skewness. Nevertheless, all values were asymmetrical, but Erongo, Omusati, Oshikoto, and Kunene regions were closer to the symmetrical distribution of zero (0) with Skp coefficient values of −0.08, −0.07, −0.02, and 0.11, respectively. Meanwhile there was a significant clinical difference between the values (*p* < 0.05) as shown in Figure 2.

## 4. Discussion

Indirect method and non-parametric percentile analysis were used to determine the reference range of 78,477 individuals (44,846 females and 33,631 males) representing the Namibian population. Overall, the reference range for the entire subjects was 51–91 g/L, as shown in Table 1. The established reference range recorded in this study is different from the reference ranges currently in use across Namibia’s clinical and pathological institutes, such as NIP, Nampath, and Clinical Laboratory Services (CLS), with the TSP reference range of 61–79 g/L, 60–80 g/L, and 57–80 g/L, respectively. The differences between the reference ranges could be due to the difference in the population race and methods used. In a study conducted in Cameroon to determine the reference ranges of clinical analytes of which TSP was one of them, the TSP reference range of 45–107 g/L was recorded [14]. Meanwhile, this range was broad, which is similar to the range established in this study (51–91 g/L) when compared with the narrow range recommended by the Center for Disease Control and Prevention (CDC) (60–80 g/L). This study used the indirect method while others used the direct method to determine the reference range. The difference in the sample size and the age distribution among the samples can also be a contributing factor to the differences in the reference ranges [3,7]. 

Reference intervals were also determined according to gender (female and male) and there was clinical significance in the difference between the mean values (*p* < 0.05) of the two genders considered. Despite the clinically significant difference in the mean values, the reference interval for males was 51–92 g/L while a close margin of 51–91 g/L was recorded for females; thus, showing that the male gender had a wider range than the female gender as shown in Table 2. This is in line with a study conducted in Pakistan by Ibrahim et al. [15], which determined the TSP reference ranges for males and females in the Karachi region. The reference range for males was 64–80 g/L, while the range for females was 65–78 g/L. However, it was in contrast to a study reported in Rwanda by Gahutu and Wane [16] with the reference range for males recorded as 63–84 g/L and 65–85 g/L for females, using the direct and non-parametric testing (percentile) methods. These studies, therefore, corroborate the fact that gender difference can also affect the TSP levels in the body as females and males have different metabolic mechanisms that the body uses for the production and the processing of the proteins in the body. Also, different proteins are produced in the bodies according to the gender and they are produced at different levels in both males and females [2]. The difference of the reference ranges could also be due to the difference in methods, geographical factors, or the normal physiological factors that take place in the body at specified times, e.g., hormonal regulation (puberty and ovulation) [17].

The data was divided into different age groups and their mean, standard deviation, skewness, kurtosis, and the percentile values are shown in Table 3. ANOVA between the mean of various age groups revealed a significant difference between the mean values (*p* < 0.05). The <1 year age group has a smaller mean (reference range = 48–83.25 g/L) compared to the rest of the age groups, meanwhile at 1–5 years of age, the TSP mean started to increase with a reference range of 48–85.55 g/L (2.5–97.5 percentiles) and a peak at 36–45 years of age (reference range = 54–93 g/L) until the 56–65 years age group (reference range = 54–91 g/L). Subsequently, a noticeable decrease was observed from >65 years age group (51–89 g/L). This is in support of a study conducted in the United Kingdom by Weaving et al. [18] on the evaluation of age and gender variations for albumin; the major constituent of TSP, using the indirect and non-parametric testing methods to determine the reference ranges. The age group of 1–5 years had an albumin level of 43 g/L and there was an increase in the albumin values until the 36–40 years age group. The albumin values started to decrease from the age group of 41–45 years (42 g/L). The current study was also in agreement with the study performed in Pakistan with the age group of <1 year having the minimum TSP reference range of 58–68 g/L and an increase from the age group of 1–3 years (54–76 g/L) to the age group of 40–49 years (66–87 g/L) and >50 years (66–89 g/L) [15]. All these studies generated results with similar outcomes or trends and therefore pointing to the fact that the liver is the major producer of proteins and in infancy and early childhood, the liver is not fully developed; hence there is a reduction in the normal functions of the liver and the protein produced. As the person grows, the liver also develops and can perform its normal production of proteins. However, as individual ages (old age), there is atrophy of the liver and because of the reduced function of the liver, the production of protein in the liver declines [19]. Children also tend to have low total plasma because of the type of diet intake while the adolescents have elevated protein levels because of the excessive production of hormones during puberty. Hormones are made up of proteins and contribute to the TSP in the body [2].

This study also focused on determining the TSP reference ranges according to the 14 regions in Namibia. Ethnicity and geographical location of subjects contribute to the total protein levels in the blood. The statistical values (mean, standard deviation, skewness, kurtosis, and percentile) are shown in Table 4. A comparison of the mean of the different regions was assessed and a significance statistical difference (*p* < 0.05) was observed between the regions. The mean was different from region to region with Ohangwena region observed to be the lowest with a reference range of 50–89 g/L while the Hardap region had the highest with a reference range of 53–92.7 g/L. Alemnji et al. [14] determined TSP reference range of Cameroonians per residential area (urban and rural). The reference range established for urban residents was 45.3–107.8 g/L while that of rural residents was 58.4–103.8 g/L. Eunjung et al. [5] also determined the albumin reference ranges for four races and the European, African, Hispanics, and Asians had a reference range of 39–51 g/L, 38–49 g/L, 39–51 g/L, and 40–51 g/L, respectively. Not only does geographical location play a role, ethnicity and diet can also contribute to the differences in the reference ranges. De Waal-Miller [20] determined reference ranges for Namibians using seven laboratory tests which were hemoglobin, urea, creatinine, glucose, cholesterol, triglyceride, and uric acid, the reference ranges showed a significant difference from the ranges used in NIP. It also showed that there were differences in the levels of fasting and random glucose, cholesterol, triglyceride, and uric acid in the different age groups and regions in Namibia [20].

One of the factors that are affecting the establishment of reference ranges is the difference in operating conditions. Different laboratories use different methods and equipment in the running of tests. Sample collection and subject preparation can also affect the reference range because it affects the levels of the protein that will be measured in the blood sample. Sample storage is also a contributing factor to protein level measurement [21,22]. Although the direct method is convenient, it is affected by the criteria of selection of the population subjects. Numerous factors matter when selecting the population to be used such as age, gender, body mass, and height. It is also very difficult to determine whether the person recruited to participate in the study is healthy and there are several variations in specific laboratory parameters that can affect the reference intervals in certain populations such as hematologic (e.g., red blood cell and white blood cell components) and clinical chemistry parameters (e.g., alanine aminotransferase and aspartate aminotransferase) [23]. The indirect method may also affect the determination of reference range in that the population being used may as well contain some unhealthy subjects. However, there are computer programs designed to eliminate the outliers [24] and the detailed analyses of the TSP range therefore obtained in this study are essential as they offer a basis of diagnostic or prognostic biomarkers in addition to an understanding into the mechanisms of disease progression [25].

## 5. Conclusions

This study reports on the TSP reference interval that was established according to gender, age groups, and different regions in Namibia. The overall TSP reference interval determined was 51–91 g/L, which is different from the reference ranges used in Namibia. The study also focused on determining the significance of gender, age, and ethnicity which was generally represented by region. The reference range for female and male were 51–91 g/L and 51–92 g/L, respectively. Meanwhile a TSP range of 48–85.55 g/L (<1–5 years), 52–91 g/L (19–25 years), 54–93 g/L (36–45 years), and 51–89 g/L (>65 years) were recorded within the different age groups. A TSP range of 53.00–92.00 g/L was noted in Erongo, Zambezi, Hardap, Kavango East, while 50.00–89.00 g/L and 54.00–91.00 g/L were recorded in Ohangwena and Omusati respectively. There was a significant statistical difference (*p* < 0.05) in reference ranges for each category (gender, age groups, and regions). Meanwhile, with a high number (78,477) of samples or participants and the successful removal of outliers with most skewness coefficient falling within the acceptable values of ±0.5 and some getting closer to symmetrical distribution (0); hence, a credible reference range recorded. To reduce the risk of inaccuracy, we therefore recommend based on our findings that about 20 samples from the healthy individuals should be used by clinical laboratory to verify the TSP reference ranges that have been determined and currently in use.

## Figures and Tables

**Table 1:** Total serum protein range and symmetrical statistic of the data.

Measurable Parameters	Values
**Percentiles**	(g/L)
2.5	51.00
5	54.00
95	88.00
97.5	91.00
**Central tendency and dispersion**	
N*	78,477
Mean	72.58
Median	73.00
Std. Deviation	9.925
**Asymmetry and existence of outliers**	
Skewness	−0.229
Std. Error of Skewness	0.009
Kurtosis	−0.159
Std. Error of Kurtosis	0.017

*N, number of samples or participants.

**Table 2:** Gender-based total serum protein range and its symmetrical distribution.

Measurable Parameters	Female	Male
**Percentiles**	(g/L)	(g/L)
2.5	51.00	51.00
5	54.00	55.00
95	88.00	89.00
97.5	91.00	92.00
**Central tendency and dispersion**		
N*	44,845	33,632
Mean	72.27	72.99
Median	73.00	74.00
Std. Deviation	9.796	10.080
**Asymmetry and existence of outliers**		
Skewness	−0.215	−0.253
Std. Error of Skewness	0.012	0.013
Kurtosis	−0.129	−0.191
Std. Error of Kurtosis	0.023	0.027

*N, number of samples or participants.

**Table 3:** Age group and symmetrical distribution of total serum protein range.

Age Group	N*	Mean	Median	Standard Deviation	Skewness	Kurtosis	Percentiles (g/L)			
							2.5	5	95	97.5
<1	2549	61.59	58.00	9.503	0.688	0.141	48.00	48.00	79.00	83.25
1–5	4257	67.62	67.00	9.155	−0.062	−0.100	49.00	51.00	82.00	85.55
6–12	2709	71.93	73.00	8.747	−0.299	0.379	52.00	55.00	85.00	88.00
13–18	3152	72.35	73.00	9.769	−0.238	−0.185	51.83	54.00	88.00	91.00
19–25	8027	72.82	74.00	9.927	−0.251	−0.182	52.00	55.00	88.00	91.00
26–35	18,003	73.23	74.00	10.033	−0.251	−0.190	52.00	55.00	89.00	92.00
36–45	13,243	74.40	75.00	9.599	−0.227	−0.031	54.00	57.00	90.00	93.00
46–55	9867	74.62	75.00	9.322	−0.336	0.219	53.00	57.00	89.00	92.00
56–65	7420	73.64	74.00	9.052	−0.281	0.176	54.00	57.00	88.00	91.00
>65	9250	71.06	72.00	9.361	−0.151	−0.152	51.00	54.00	86.00	89.00

*N, number of samples or participants.

**Table 4:** Regional reference range distribution of total serum protein.

Region	N*	Mean	Median	Standard Deviation	Skewness	Kurtosis	Percentiles			
							2.5	5	95	97.5
Otjozondjupa	2925	71.96	73.00	9.858	−0.123	−0.214	51.00	55.00	88.00	91.00
Khomas	26,014	72.80	74.00	10.227	−0.342	−0.169	51.00	53.00	88.00	91.00
Oshikoto	6021	71.95	72.00	9.952	−0.137	−0.249	51.00	54.00	88.00	91.00
Oshana	11,887	72.07	73.00	9.710	−0.279	−0.099	51.00	54.00	87.00	90.00
Erongo	2952	73.76	74.00	9.428	−0.235	0.021	53.83	57.00	89.00	92.00
Ohangwena	4114	69.78	69.00	9.728	−0.013	−0.204	50.00	53.00	85.00	89.00
Kavango West	979	71.56	71.00	9.979	−0.054	−0.266	51.50	54.00	89.00	91.00
Karas	1862	72.25	73.00	9.756	−0.089	−0.162	52.00	56.00	88.00	91.00
Zambezi	2523	72.86	72.00	9.868	−0.082	−0.241	53.00	56.00	89.00	92.00
Hardap	1532	74.45	75.00	10.060	−0.314	−0.223	53.00	56.00	89.35	92.67
Kunene	1681	72.34	72.00	9.684	−0.011	0.028	52.00	55.00	89.00	92.00
Kavango East	12,353	73.41	74.00	9.641	−0.146	−0.132	53.00	57.00	89.00	92.00
Omusati	2986	73.79	74.00	9.064	−0.215	0.051	54.00	58.00	88.00	91.00
Omaheke	648	70.51	68.50	9.981	−0.024	−0.251	51.00	53.00	87.55	91.00

*N, number of samples or participants.
